# CD6 attenuates early and late signaling events, setting thresholds for T-cell activation

**DOI:** 10.1002/eji.201040528

**Published:** 2011-09-28

**Authors:** Marta I Oliveira, Carine M Gonçalves, Mafalda Pinto, Stéphanie Fabre, Ana Mafalda Santos, Simon F Lee, Mónica A A Castro, Raquel J Nunes, Rita R Barbosa, Jane R Parnes, Chao Yu, Simon J Davis, Alexandra Moreira, Georges Bismuth, Alexandre M Carmo

**Affiliations:** 1 Group of Cell Activation and Gene Expression, IBMC-Instituto de Biologia Molecular e CelularPorto, Portugal; 2 ICBAS-Instituto de Ciências Biomédicas Abel Salazar, Universidade do PortoPorto, Portugal; 3 Institut National de la Santé et de la Recherche MédicaleParis, France; 4 Institut Cochin, Université Paris Descartes, Centre National de la Recherche Scientifique (UMR 8104), Equipe labellisée par la Ligue Nationale contre le CancerParis, France; 5 MRC Human Immunology Unit, Weatherall Institute of Molecular Medicine, Nuffield Department of Clinical Medicine, University of OxfordOxford, UK; 6 Department of Medicine, Division of Immunology and Rheumatology, Stanford University School of MedicineStanford, CA, USA

**Keywords:** Cellular activation, Cell-surface molecules, Inhibitory molecules, Signal transduction, T cells

## Abstract

The T lineage glycoprotein CD6 is generally considered to be a costimulator of T-cell activation. Here, we demonstrate that CD6 significantly reduces early and late T-cell responses upon superantigen stimulation or TCR triggering by Abs. Measuring calcium mobilization in single cells responding to superantigen, we found that human T cells expressing rat CD6 react significantly less well compared with T cells not expressing the exogenous receptor. When the cytoplasmic domain of rat CD6 was removed, calcium responses were recovered, indicating that the inhibitory properties of CD6 are attributable to its cytoplasmic domain. Calcium responses, and also late indicators of T-cell activation such as IL-2 release, were also diminished in TCR-activated Jurkat cells expressing human CD6, compared with CD6-deficient cells or cells expressing a cytoplasmic deletion mutant of human CD6. Similarly, calcium signals triggered by anti-CD3 were enhanced in human T lymphocytes following morpholino-mediated suppression of CD6 expression. Finally, the proliferation of T lymphocytes was increased when the CD6–CD166 interaction was blocked with anti-CD166 Abs, but inhibited when anti-CD6 Abs were used. Our data suggest that CD6 is a signaling attenuator whose expression alone, i.e. in the absence of ligand engagement, is sufficient to restrain signaling in T cells.

## Introduction

CD6 is an integral membrane protein expressed on peripheral T lymphocytes and medullary thymocytes 1. CD6 is likely to be involved in the regulation of T-cell development and activation 2, and to have an important role in the context of human inflammatory responses and cellular expansions, given that the CD6 gene is strongly associated with susceptibility to multiple sclerosis (MS) according to genome-wide association studies and meta analyses 3, 4. Anti-CD6 IgM Abs have been used in clinical trials with MS patients, although they are yet to have a clinical impact 5. Given this level of clinical interest, however, it is of considerable importance that the signaling pathways regulated by CD6 are better characterized.

CD6 has long been regarded as a costimulatory molecule largely because Ab-mediated CD6 cross-linking potentiates proliferative T-cell responses 6, 7, and also because soluble, monomeric CD6 present in antigen (Ag)-primed mixed lymphocyte reactions inhibited Ag-specific T-cell responses 8–10. Upon TCR engagement, CD6 is phosphorylated on tyrosine residues suggesting that interactions with SH2 domain-containing intracellular mediators very likely occur 11. The cytoplasmic domain of CD6 additionally contains multiple potential binding sites for SH3 domain-containing signaling effectors 12. Accordingly, the rat homologue of CD6 has the capacity to associate with protein tyrosine kinases of different families, such as the Src-family kinases Lck and Fyn, the Syk family kinase ZAP-70, and the Tec-family kinase Itk 13. Beyond this neither the signaling pathways directly triggered by CD6 stimulation nor even the character of the resulting signals is well understood.

Recent studies have shown that CD6 associates with SLP-76, a positive regulator of T-cell activation 9, and with the scaffolding protein syntenin-1, possibly coupling CD6 to the cytoskeleton and other signaling effectors 14. CD6 has also been shown to associate at the surface of T cells with the structurally related receptor CD5 13, 15, an inhibitor of T-cell responses 16–18. CD5 and CD6 exhibit a shared pattern of expression during ontogeny, and the possibility of overlapping effects of these two proteins on repertoire selection has been previously suggested 19. Moreover, stimulation of rat T cells with anti-CD6 Abs enhanced the tyrosine phosphorylation of CD5 13, suggesting that CD6 triggering may increase the inhibitory potential of CD5.

The molecular basis of the interaction between CD6 and its physiological ligand, CD166, has been comprehensively studied 2. CD166 is expressed on cortical and medullary thymic epithelial cells, monocytes and transiently on activated leukocytes, indicating that the contact between CD6 and CD166 may be important during thymic selection and during and after the activation of mature T cells 9, 20. CD6 is also reported to accumulate at the immunological synapse upon T cell–APC conjugate formation 8, with the interaction depending on the binding of APC-expressed CD166 to the third scavenger receptor cysteine-rich extracellular domain of CD6 21.

We have investigated the signaling role of CD6 during Ag presentation and in vitro stimulation. Surprisingly, contrary to expectation based on the effects of CD6 cross-linking, which activates T cells 6, 7, 13, we consistently observe that the expression of CD6 at the surface of T cells abrogates both early and late signaling responses triggered by Ag or by direct stimulation of the TCR/CD3 complex, even in the absence of ligand binding. Our results strongly suggest that, as in the case of the evolutionarily related molecule CD5, CD6 is a negative modulator of T-cell activation whose expression alone at the T-cell surface is sufficient to constrain T-cell activation.

## Results

### CD6 expression inhibits calcium responses in T cells

To investigate the signaling role of CD6, we used ex vivo human T lymphocytes and induced the expression of exogenous rat CD6 (rCD6) at their cell surface. We then measured calcium responses of individual T cells, either expressing or not rCD6, interacting with superantigen (sAg)-loaded APCs. Transient manipulations of T-cell signaling molecules in this system have been useful for identifying critical parameters for T-cell activation previously 22.

**Figure 1:**
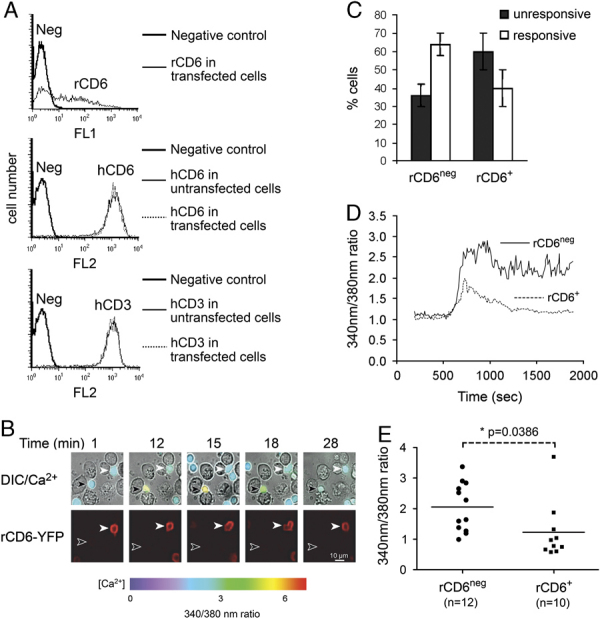
CD6 decreases the amplitude of Ca^2+^ responses. (A) Cytometric analysis of ex vivo human T cells transfected with an rCD6-YFP vector. Live cells were gated based on the Forward Scatter (FSC) and Side Scatter (SSC) profiles. The expression of rCD6 is indicated by a thin line (upper panel) and the negative control is shown (in all panels) as a thick line. Expression of hCD6, (middle panel), and human CD3 (hCD3, lower panel) is shown before (thin line) and after transfection (dashed line) of T cells with the vector. (B) Raji cells preincubated with sAg interacted with Fura-2 loaded T lymphocytes transfected with a cDNA construct encoding rCD6-YFP. Time-lapse video imaging with 20× magnification showing superimposed differential interference contrast (DIC) and calcium signals (upper panel) and rCD6 expression (lower panel) on one rCD6^−^ T cells (black arrow) and one rCD6^+^ T cell (white arrow), interacting with APCs in the same field. Scale bar=10 μm. (C) Percentage of rCD6^−^ or rCD6^+^ T cells that increase (ratio >1.5) or not their calcium after conjugate formation with sAg-pulsed Raji cells. Bars represent the mean (±SD) values of three separate experiments with at least 30 individual cells analyzed in each. (D) Intracellular calcium was measured sequentially every 10 s in T cells interacting with sAg-pulsed Raji cells. Calcium was measured for T cells expressing (dashed line) or not (solid line) rCD6-YFP. Calcium signals were averaged from individual responses of all the cells in each set. Results are from one of the three experiments showing similar results. (E) Dot plots showing maximal calcium signals obtained in individual cells following stimulation by sAg-loaded Raji cells. Each dot represents one cell. Horizontal lines indicate mean values [rCD6^−^ (*n*=12); rCD6^+^ (*n*=10)]. The probability that the Ca^2+^ signals of rCD6^+^ T cells in response to sAg stimulation were similar to those of rCD6^−^ T cells was assessed using Students *t*-test (*p*=0.038).

Following transfection with a cDNA encoding rCD6 fused to YFP, the human T cells expressed variable levels of the chimeric protein (Fig. 1A, top panel). Endogenous human CD6 (hCD6) as well as human CD3 (hCD3) were highly expressed and their expression was unaffected by the coexpression of rCD6 (Fig. 1A, middle and bottom panels, respectively). Similarly, Raji cells (used as APCs) express human CD166 (hCD166), and so presumably in T cell–APC conjugates, T-cell surface hCD6 interacts with hCD166 expressed in Raji cells. We have considered untransfected T cells interacting with untransfected Raji the standard experimental setting, and changes that may occur following the expression of rCD6 and rCD166 should be due to specific functions of these heterologous rat molecules.

T cells were allowed to interact with sAg-loaded Raji cells, and intracellular calcium levels in individual T-cell/Raji cell conjugates were followed by fluorescence imaging. Strikingly, T cells expressing high levels of exogenous rCD6 appeared to be much less responsive to APCs than T cells that were low or negative for rCD6; a typical example is shown in Fig. 1B (see also Supporting Information movie 1). To quantitate this effect, we selected T cells expressing either high levels of rat CD6 (rCD6^+^), or cells for which rCD6 expression was not detectable in the fluorescence microscope (rCD6^−^). Sixty percent of rCD6^+^ cells failed to respond to the APCs, compared with only 36% of the rCD6^−^ cells (Fig. 1C). For the responding cells of both the rCD6^+^ and the rCD6^−^ groups, we measured the calcium signals in individual cells. On average, rCD6^+^ cells (*n*=10) displayed lower calcium signals than rCD6^−^ cells (*n*=12), and the response returned to baseline more quickly (Fig. 1D). The peak levels of the calcium responses were significantly lower for the rCD6^+^ cells than those of the rCD6^−^ cells (*p*<0.05; Fig. 1E).

**Figure 2:**
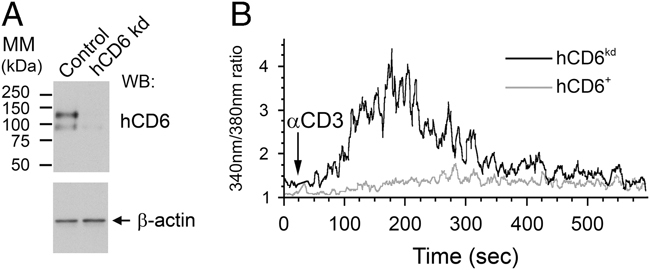
Expression of hCD6 downregulates calcium signals. (A) Purified human primary T lymphocytes were treated with specific hCD6 MOs to suppress endogenous hCD6 expression (hCD6 kd), or with control scramble morpholinos (Control). Knockdown of hCD6 was evaluated by Western blot of cell lysates, using β-actin as a loading control. Numbers on the left indicate molecular mass markers (MM) in kilodaltons (kDa). (B) Cells were incubated with calcium indicators and after stimulation with CD3 mAb (OKT3), cells expressing hCD6^+^ (grey line) and cells where hCD6 was knocked down (hCD6^kd^, black line) were evaluated for calcium mobilization by cytometry. Data are shown from one out of the three experiments with similar results.

To determine whether endogenous hCD6 would exhibit comparable inhibitory behavior in these cells, we suppressed its expression using hCD6-specific morpholino oligomers (MOs); we were successful in reducing expression by over 95% as assessed by western blotting (Fig. 2A). Using CD3 mAb (OKT3), we activated the untransfected T cells as well as T cells treated with the MOs, and measured calcium mobilization. At 1 μg/mL to activate 3×10^6^ cells/mL, OKT3 induced a very modest increase in calcium in hCD6-expressing T cells, whereas a pronounced increase in calcium signaling was observed in the hCD6-depleted T cells (Fig. 2B), confirming the observations made for the rCD6 transfections.

### Localization of CD6 at the immune synapse is not required for the inhibition of calcium signaling

**Figure 3:**
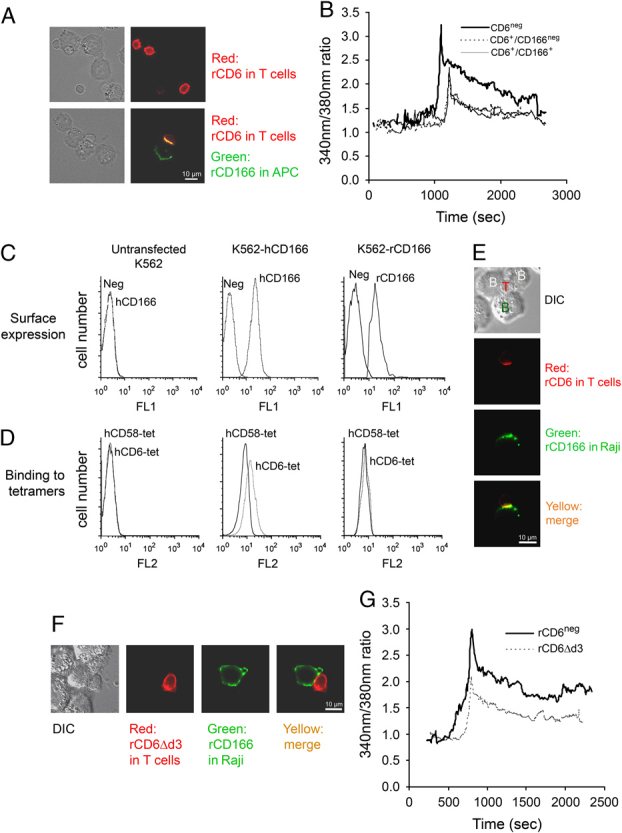
Ligand-binding interactions are required for CD6 accumulation at the immunological synapse but not for downmodulation of Ca^2+^ signaling. (A) Conjugates formed between sAg-pulsed Raji cells and rCD6-YFP-expressing T cells. Left panels: differential interference contrast (DIC) images. Upper right panel: localization of rCD6 (visualized in red) when rCD166 is not expressed on the surface of Raji cells; lower right panel: localization of rCD6 (red) and rCD166 (green) when rCD166 is expressed on the surface of Raji cells. Fluorescence overlays (yellow) of rCD6-YFP and rCD166-CFP localization are shown. Magnification is 40×, scale bar=10 μm. (B) Intracellular calcium was measured in rCD6^+^ T cells establishing contacts with rCD166^+^ Raji cells (thin line, *n*=16), rCD6^+^ T cells establishing contacts with rCD166^−^ Raji cells (dashed line, *n*=11), or rCD6^−^ T cells establishing contacts with rCD166^+^ Raji cells (thick line, *n*=15). Raji cells were previously pulsed with sAg. Averaged responses shown are from the calcium profiles of individual cells from each set, from two independent experiments. (C) Expression of human CD166 in untransfected (left) or hCD166-cDNA-transfected cells (middle), and of rat CD166 in rCD166-cDNA-transfected cells (right). Cells were gated based on the expression of hCD166 or rCD166, respectively, and these gates were used for the analysis in (D). The negative control (Neg) is also shown. (D) Binding of hCD6 tetramers (hCD6-tet, gray lines) to untransfected K562 cells (left), K562-hCD166 cells (middle), and K562-rCD166 cells (right). Binding of hCD58 tetramers (hCD58-tet, thick lines) to the cells was used as negative control, as K562 cells do not express the hCD58 ligand, hCD2. Profiles of hCD58-tet overlapped with the profiles of unlabeled cells (data not shown). (E) Interactions between one T cell expressing rCD6-YFP (T in red) and three sAg-loaded Raji cells, one of which expresses rCD166-CFP (B in green) and two that do not express rCD166 (B in white). Fluorescence overlays (yellow) of rCD6 and rCD166 localization are shown. Magnification is 40×, scale bar=10 μm. (F) Raji cells transfected with a rCD166-CFP vector were pulsed with a sAg mix before incubation with ex vivo T cells expressing rCD6Δd3, a naturally occurring isoform devoid of the rCD166 binding domain. Cells were fixed after 45 min of interaction. Localization of rCD166-CFP (green) and rCD6Δd3-YFP (red) as well as fluorescence overlays (yellow) are shown with a magnification of 40×. Scale bar=10 μm. (G) Intracellular calcium was measured sequentially every 10 s in T cells interacting with sAg-pulsed Raji cells. Calcium mobilization was measured for T cells lacking rCD6 (rCD6^−^, solid line) or T cells expressing rCD6Δd3 (dashed line), interacting with rCD166-expressing Raji cells. Averaged responses were determined from 12 rCD6^−^ T cells and 16 rCD6Δd3-expressing T cells interacting with sAg-loaded Raji cells from two independent experiments.

In order to promote rCD6 localization to the immune synapse (IS), we transfected Raji cells with a cDNA-encoding rat CD166 (rCD166) fused to CFP. As expected, when Raji cells lacked expression of rCD166, rCD6 in T cells was distributed homogeneously over the cell surface and there was no particular enrichment at the IS (Fig. 3A, upper panels). In contrast, for Raji cells expressing rCD166, both the rCD6 expressed by the T cells and the rCD166 expressed by the Raji cells accumulated in the contact area (Fig. 3A, bottom panels). Measurements of calcium mobilization in rCD6^+^ cells establishing contacts with Raji cells expressing rCD166 (rCD166^+^; *n*=16) or not expressing rCD166 (rCD166^−^; *n*=11) revealed no differences in signaling for T cells interacting with the two sets of target cells (Fig. 3B and Supporting Information Fig. 1A). This clearly indicates that the localization of rCD6 at the IS per se is not required in order for it to suppress calcium signaling.

### Human CD6 does not cross-react with rat CD166

A potential complication in the interpretation of these experiments is that hCD6, whose movement is not tracked, could interact with the rCD166 transfected into the Raji cells. Since mouse CD166 is reported to bind human as well as mouse CD6 23, we investigated whether cross-species binding between human and rat forms could be occurring in our heterologous system. For this purpose, we expressed the extracellular region of hCD6 and assembled phycoerythrin-labeled CD6 tetramers that were then used to screen for CD6 binding to rat or human CD166-expressing cells. K562 cells, of human origin, do not express CD166, and were transfected with cDNAs encoding rat or human CD166 (Fig. 3C). Although, as expected, the CD166^−^ K562 cells did not bind the hCD6 tetramer (Fig. 3D, left panel), hCD166-expressing K562 cells were clearly recognized (Fig. 3D, middle panel). In contrast, rCD166-expressing K562 cells were essentially negative for binding the hCD6 tetramers (Fig. 3D, right panel). Additional evidence that cross-species binding does not mediate CD6 translocation in our system is obtained from the observation that when rCD6-expressing cells contacted Raji-expressing rCD166 versus rCD166^−^ Raji, there was a clear preference for the accumulation of rCD6 in synapses with Raji-expressing rCD166 (Fig. 3E).

### A ligand nonbinding form of CD6 suppresses calcium responses

The experiments described above suggest that the interaction of CD6 with its ligand does not enhance its signaling suppressing effects. Further confirmation was obtained by expressing, on ex vivo human T cells, ratCD6Δd3 (rCD6Δd3), a natural occurring isoform lacking domain 3, the CD166-binding domain of rCD6 21. T cells expressing rCD6Δd3 fused to YFP were allowed to form conjugates with Raji cells expressing rCD166 and we observed that rCD6Δd3 failed to accumulate at the immune synapse according to fluorescence microscopy-based analysis (Fig. 3F). Measurements of the calcium signals arising in T cells that express rCD6Δd3 (*n*=16) and interact with Raji cells expressing rCD166, versus cells lacking the construct (rCD6^−^; *n*=12) indicate once again that the expression of rCD6Δd3 downmodulates calcium signals (Fig. 3G and Supporting Information Fig. 1B). This supports our preceding observations, i.e. that it is the expression of CD6 per se, rather than its interactions with its ligand or its synapse localization, that is responsible for the inhibition of calcium signaling.

### The inhibitory effects of CD6 depend on its cytoplasmic domain

**Figure 4:**
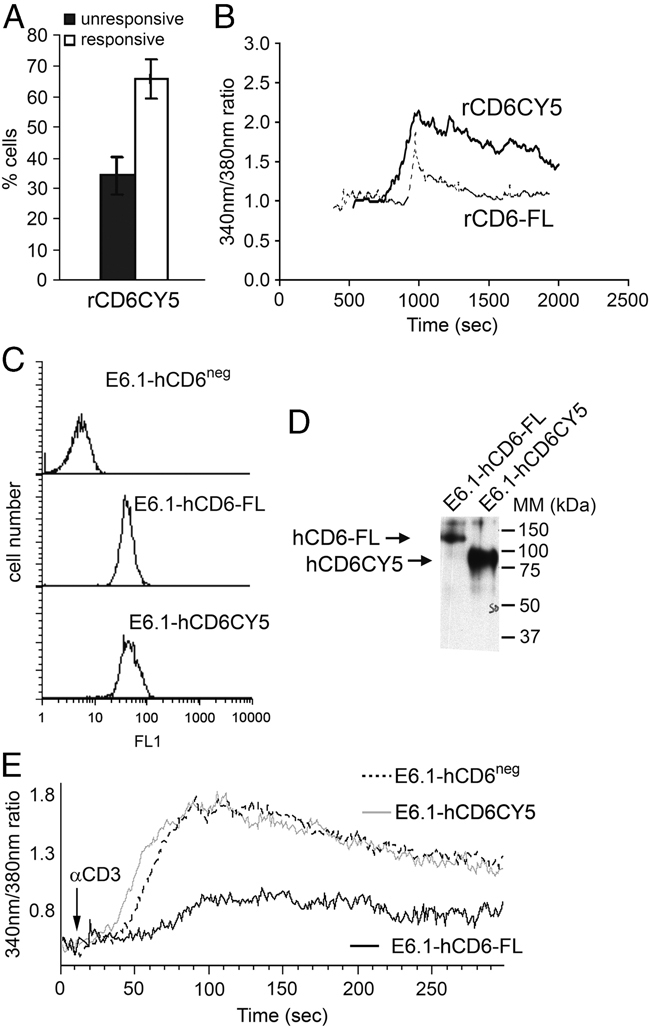
The inhibitory role of CD6 is dependent on its cytoplasmic domain. (A) Percentage of calcium responsive (ratio >1.5) or unresponsive (ratio <1.5) T cells expressing a rCD6 cytoplasmic deletion mutant (rCD6CY5-YFP), after conjugate formation with sAg-pulsed Raji cells. Bars represent the mean (±SD) values of three separate experiments with at least 30 individual cells analyzed in each. (B) Calcium signals were acquired sequentially every 10 s from rCD6CY5-YFP T cells (solid line) versus rCD6^+^ T cells (expressing full-length rCD6, dashed line), interacting with sAg-pulsed Raji cells. Averaged responses were determined from 15 rCD6^+^ T cells and 10 rCD6CY5-expressing T cells interacting with sAg-loaded Raji cells. Results are from one of two experiments, both gave similar results. (C) Surface expression of hCD6 in untreated E6.1 Jurkat cells (top), in Jurkat cells following transfection with a cDNA construct encoding full-length hCD6 (hCD6-FL) (middle), and in Jurkat cells following transfection with a cDNA construct encoding a cytoplasmatic deletion mutant of hCD6 (hCD6CY5) (bottom). Live cells were gated based on the FSC and SSC profiles. (D) E6.1-hCD6-FL and E6.1-hCD6CY5 cells were cell-surface biotinylated, lysed in detergent, and CD6 species were immunoprecipitated using MEM-98 mAb and run on SDS-PAGE. (E) E6.1 Jurkat cells expressing hCD6-FL (black lines) or hCD6CY5 (gray lines), and CD6^−^ cells (dashed lines) were preincubated with the calcium indicators Fluo-3 and Fura-red and, upon anti-CD3 stimulation, total intracellular calcium levels were monitored by cytometry during 5 min. Results are from one of three experiments, all gave similar results.

We addressed the role of the cytoplasmic domain of CD6 using an engineered isoform of rCD6 lacking its cytoplasmic domain, rCD6CY5 fused to YFP, which was expressed in ex vivo human T cells. rCD6CY5 moved to the synapse when the T cells were allowed to form conjugates with rCD166-expressing Raji cells (data not shown). Calcium signals were measured in individual cells conjugated with rCD166^+^ Raji cells, and we found that the fraction of T cells expressing rCD6CY5 that increased their calcium levels following conjugate formation (i.e. ∼66%; Fig. 4A) closely matched that of rCD6^−^ cells (Fig. 1C). For the responding T cells expressing rCD6CY5 (*n*=10), we measured calcium signals and compared these with signals from responding T cells expressing full-length rCD6 (*n*=15; Fig. 4B and Supporting Information Fig. 2). The results indicate that rCD6-expressing cells displayed lower and more transient calcium signals than cells expressing rCD6CY5, and thus the inhibitory effects of CD6 can be assigned to its cytoplasmic tail.

We have also addressed the role of hCD6, and of its cytoplasmic domain, by the analysis of calcium fluxes in whole-cell populations. E6.1 Jurkat cells, which already express very low levels of hCD6, were FACS-sorted and gated on the most-negative hCD6 cells, E6.1-hCD6^−^, which were used for our assays. These E6.1-hCD6^−^ cells were stably reconstituted with either full-length hCD6, to give E6.1-hCD6-FL, or the human cytoplasmic deletion mutant, to yield E6.1-hCD6CY5 (Fig. 4C). Before each experiment, E6.1-hCD6-FL and E6.1-hCD6CY5 were FACS sorted so that the levels of hCD6-FL and hCD6CY5, respectively, were equivalent. We confirmed by immunoprecipitation of hCD6 from surface biotinylated cells and Western blotting, that E6.1-hCD6-FL cells expressed only the full-length hCD6 protein (∼130 kDa) and that E6.1-hCD6CY5 cells expressed only the cytoplasmic domain-deleted form of hCD6 (∼90 kDa; Fig. 4D). Calcium responses upon CD3 stimulation (OKT3 at 1 μg/mL using 3×10^6^ cells/mL) were similarly high in both hCD6^−^ cells and cells expressing hCD6CY5, whereas cells expressing full-length hCD6 exhibited much poorer signaling capabilities (Fig. 4E), confirming the cytoplasmic domain dependence of calcium signaling by both human and rCD6.

### CD6 modulates late signaling responses

**Figure 5:**
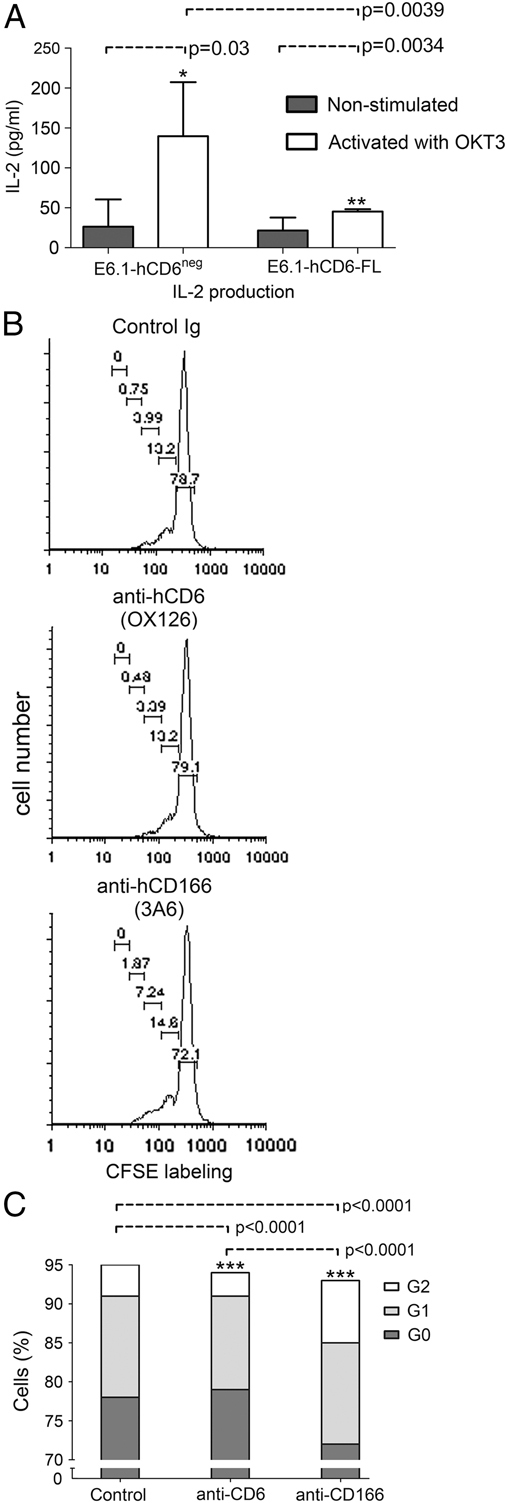
CD6 limits late signaling events. (A) IL-2 production (pg/mL) by hCD6^−^ versus hCD6-expressing (hCD6-FL) E6.1 cells, treated (white bars) or not treated (black bars) with OKT3. Data shown are mean+95% confidence intervals from three independent experiments. The probability that the production of IL-2 by hCD6^−^ and hCD6FL E6.1 cells was similar, with and without anti-CD3 mAb stimulation, was assessed using Studentŉs *t*-test (for *p*–values, see graph). (B) CFSE-preincubated human PBLs were allowed to interact for 6 days with sAg-pulsed Raji cells expressing endogenous human CD166, in the presence of blocking (anti-CD166-3A6 and anti-CD6-OX126) or nonblocking (control) Abs. Cell division was determined by the extent of CFSE dilution. (C) Effect of CD6 expression on cell proliferation. An equal number of cells were analyzed by FACS following CFSE labeling. Stacked bars represent the mean number of cells in each generation (G0–G2) from three independent experiments. The probability that the proportion of cells in G0–G2 was the same in hCD6-FL-expressing cells in the presence of control or blocking (anti-CD166-3A6; [*p*<0.0001], anti-CD6-OX126; [*p*<0.0001]) Abs was assessed using the χ^2^-test. ^***^p<0.0001.

We next evaluated whether CD6 was also able to exert an inhibitory effect on downstream functional responses, such as IL-2 production. To address this, E6.1-hCD6^−^ or E6.1-hCD6-FL cells were stimulated with OKT3, or with a negative control Ab. Following stimulation, cells expressing hCD6 produced three-fold less IL-2 than the parental cells (Fig. 5A). We also assessed the role of CD6 in untransfected ex vivo T cells interacting with APCs, by testing whether blocking the CD6–CD166 interaction altered the proliferative behavior of the T cells. Ex vivo lymphocytes were allowed to interact with sAg-loaded Raji cells, which express endogenous human CD166, in the presence or absence of blocking anti-human CD166 and CD6 Abs 21. As shown in Fig. 5B and C, preincubating the Raji cells with the CD166 Ab resulted in enhanced cell proliferation such that the fraction of CD6–CD166 blocked T cells completing two rounds of proliferation after 6 days of activation was double that of control cells. In contrast, when we blocked the interaction using a CD6 Ab, cellular proliferation was inhibited, suggesting that binding of the mAb to CD6 induces inhibitory signaling in the T cells (Fig. 5C). We concluded that CD6 constrains both short-term and long-term signaling events in T cells.

## Discussion

In this report, we present evidence that, like CD5, CD6 is capable of generating inhibitory signals in T lymphocytes. Our main findings are (i) that transfected rCD6 and hCD6 reduce Ca^2+^ responses in human T cells and a T-cell line, respectively, and that the suppression of hCD6 expression increases these responses; (ii) that the ligand-dependent localization of CD6 to the synapse is neither required for these effects nor, surprisingly, further enhances them; (iii) that the cytoplasmic domains of human and rCD6 are responsible for these inhibitory effects; and (iv) that, in addition to receptor proximal signaling, CD6 expression suppresses longer term events such as cytokine secretion and T-cell proliferation. We conclude from these experiments that CD6 has an important role in establishing thresholds for T-cell activation.

CD6 was previously regarded to be a costimulatory receptor however, this assumption was largely built on reports by ourselves and others in which CD6 was cross-linked together with the TCR using Abs 6, 7, 13. An explanation for these observations is that CD6 associates with protein tyrosine kinases such as Lck, Fyn, and ZAP-70 13, and it could be expected that mAb-induced coaggregation of CD6 with the TCR might promote the Lck/Fyn-mediated phosphorylation of CD3 ITAMs, leading to signaling. CD5 was originally proposed to be a costimulatory molecule for similar reasons 24, 25, but was subsequently shown to inhibit T-cell activation and thymocyte selection 18.

More recent studies have shown that blocking the CD6–CD166 interaction with soluble recombinant ligands or Abs reduces T-cell activation and proliferation following the interaction of T cells with APCs 8–10, 26, supporting a costimulatory role for CD6. In these experiments, however, the blocking Abs and recombinant proteins used significantly diminished the number of T cell–APC conjugates that formed, implying that the observed inhibitory effects were not simply due to the blockade of costimulatory processes. Zimmerman et al. 10 observed that a nonblocking CD6 mAb (M-T605) that targets domain 1 of CD6 rather than the ligand-binding domain 3, and therefore ought not to block the CD6/CD166 interaction, is a potent inhibitor of T-cell proliferation. Almost complete inhibition of proliferation was also observed in cultures in which soluble CD166-Fc was used as a blocking agent, whereas the inhibition was much less pronounced when a CD6–Fc fusion protein was used. Similarly, Montero and colleagues showed that in the cultures of T cells interacting with plate-bound recombinant CD166 and CD3 mAb, the inclusion of a nonblocking CD6 domain 1-binding Ab markedly reduced intracellular phosphorylation and strongly inhibited T-cell proliferation 27. Given that only the reagents that interact directly with the CD6 molecule completely abolish T-cell activation, it seems reasonable to propose that in these experiments engaging CD6 delivers a negative signal to the T cells, as is now more exhaustively documented in the present experiments.

However, it is also possible that CD6 engagement strengthens T-cell activation, albeit indirectly. The interaction of CD6 with CD166 is fairly strong 9, and is likely to increase cellular adhesion and in this way enhance T-cell responses. Thus, a dual function for CD6 could be envisaged, whereby the balance of positive (stronger adhesion) and negative (inhibitory signaling) processes fine-tunes T-cell activation. Hassan et al., while showing that the interaction between CD6 and CD166 is important for T-cell activation, have reported that an increase in the level of CD6 expression made T cells less responsive 26, in agreement with our present observations. The overall balance between inhibitory and activatory signals, including the strength of the signals initiated by the TCR, is likely to dictate the outcome of the activation process.

The molecular mechanisms through which CD6 constrains signaling are not yet determined, but it is possible that CD6 functions in a similar way of CD5, coupling with inhibitory tyrosine phosphatases, e.g. SHP-1, or modulating the activity of signaling-enhancing tyrosine kinases 28, 29. Alternatively, given that CD6 associates with several kinases, it might sequester these kinases away from the TCR apparatus or it could function analogously to the IgSF glycoprotein CD2, which transduces mitogenic signals via its association with lipid rafts and the tyrosine kinases Lck and Fyn 30, 31, and inhibitory signals via its association with CD5 32–34. Since CD5 and CD6 also associate at the surface of T cells and one major effect of CD6 stimulation is the phosphorylation of CD5 13, it is feasible that CD5 can integrate or transform the signals generated by the triggering of CD6.

A striking finding of the present study is that CD6 appears to be capable of strong inhibitory signaling in the absence of ligand. Our findings suggest that CD6 is a general attenuator of T-cell activation, whose level of expression at the plasma membrane, rather than its interactions with ligand or localization at the immunological synapse, determines the amplitude of the signal. At present, we are unable to offer an explanation for why the localization of CD6 to the synapse does not induce stronger inhibitory signaling. A correlation between the levels of CD6 expression and the heightened inhibition of cell responsiveness has been proposed previously 9. However, given that very few T cells do not express CD6, finer-grain regulation may be achieved by the generation of CD6 intracellular isoforms produced at distinct stages of activation or differentiation 12, 35, 36. Since, as we show, the cytoplasmic domain is crucial for the inhibitory role of CD6, variation in its composition may translate into different associations with effector molecules that could very conceivably fine-tune activation thresholds in T cells.

Nevertheless, the full-length isoform seems to be the most abundant, and hence at least at some stages of immune responses, CD6 will display some capacity for downmodulating activation signals as was shown for the full-length constructs used in the present study. As the cytoplasmic part of CD6 is highly tyrosine phosphorylated after T-cell activation, the example of CD5 could be followed 16, 17, with a systematic and careful analysis of the various tyrosine residues present within the domain of CD6 undertaken. The observation that, like CD6, CD5 also attenuates signaling in T and B cells stimulated via their physiological receptors in a ligand independent manner, suggests that CD6 is not alone in its capacity to establish activation thresholds via its expression per se, and that this could be a general feature of inhibitory signaling proteins.

## Materials and methods

### Plasmid constructs

rCD6FL/pEYFP-N1, rCD6Δd3/pEYFP-N1 lacking the ligand-binding domain of CD6, and a cytoplasmic deletion mutant, rCD6CY5/pEYFP-N1, as well as rCD166/pECFP-N2 have been described previously 21.

To produce a truncated form of hCD6, the expression vector encoding full-length hCD6, CD6/pBJ-neo 12, was used as a template for the amplification of a PCR fragment, with a forward primer at the beginning of the transmembrane domain (5′-CAAGGAATCTCGGGAGCTAATGCTCC-3′) and a reverse primer including the first tyrosine residue codon of the cytoplasmic tail, followed by a stop codon and a *Xba*I restriction site (5′-ACTATCTAGACTACTACTCGAGCGGCCGCCACTGTGCTGGATATCTGCAGAATTAATATT-3′). The PCR product was then subcloned into CD6CY5/pcDNA3.1/CT-GFP-TOPO 21, in the *Eco*RI-*Xba*I restriction sites.

Human CD166 cDNA was obtained from activated PBMC, using forward (5′-TAGTAGAAGCTTATGGAATCCAAGGGGGCCAG-3′) and reverse primers (5′-ACAATCACAAAACTGAAGCCCGCGGTAGTAG-3′) in a PCR reaction, and cloned into the pECFP-N2 vector in *Hind*III*-Sac*II restriction sites.

### Cells and transfections

Cells lines Raji 37, Jurkat E6.1 38, K562 39, and CHO-K1 40 were maintained in RPMI 1640 supplemented with 10% FCS, 1 mM sodium pyruvate, 2 mM l-glutamine, penicillin G (50 U/mL), and streptomycin (50 μg/mL). Human peripheral blood lymphocytes were obtained from peripheral blood of healthy donors after centrifugation over Lymphoprep (Nycomed Pharma). Human primary T cells were isolated from peripheral blood by centrifugation in a Ficoll gradient followed by negative depletion on magnetic beads (T-cell negative isolation kit, Dynal Biotech).

Transient transfections of primary T lymphocytes and of Raji cells were performed as described previously 21. Jurkat E6.1 cells were stably transfected as described previously 31. K562 cells (5×10^6^) were transfected with 50 μg of hCD166/pECFP-N2 or rCD166/pECFP-N2 and by electroporation at 500 μF and 875 V and cultured in complete RPMI supplemented with 5 mg/mL of G418 (Invitrogen) to select for positive cells.

### Flow cytometry and cell sorting

Flow cytometry was performed as described previously 33. Monoclonal Abs used were OX52 recognizing rCD6 41, MEM-98 recognizing hCD6 42, and UCHT1, recognizing human CD3 43; polyclonal Abs were rabbit anti-mouse (RAM) FITC-labeled (DakoCytomation) and rabbit F(ab′)_2_ anti-mouse RPE (Dako). Fluorescence-activated cell sorting was performed using a FACSAria (Becton Dickinson).

### Conjugate formation and fluorescence analysis

Conjugate formation and fluorescence analysis were performed as described previously 21.

### Calcium measurements

Single-cell calcium measurements were performed as described previously 22. To measure total calcium release in human T-lymphocyte populations or Jurkat cell lines, 3×10^6^ cells were loaded with the calcium indicators Fluo-3 and Fura Red (Molecular Probes) at the final concentrations of 5 and 10 μM, respectively, in complete RPMI and incubated for 45 min at 37°C. Basal calcium levels were analyzed by flow cytometry for 10 s before samples were removed, the agonist (OKT3 at 1 μg/mL) was added, and the samples monitored for calcium release during 5 min. Cells were at a concentration of 3×10^6^ cells/mL. Analysis was performed using the FlowJo software (Treestar). The ratio FL1/FL3 was derived and plotted against time. Kinetic plots are represented as mean of the FL1/FL3 ratio.

### CD6 knockdown using MOs

Knockdown of hCD6 from T lymphocytes was achieved through the transient transfection of CD6 MOs into T cells using Amaxa nucleofection. In brief, 1×10^7^ cells were centrifuged, resuspended in 100 μL of prewarmed nucleofector solution, mixed with 150 μM of MOs for CD6 (MO-CD6, 5′-GTCTGGAGCTGTCTCTGGCTGCTAC-3′, GeneTools), or 150 μM of scramble MO-control (MO-c; 5′-GTgTGCAGCTCTCTCTGCCTGGTAC-3′-fluorescein), and transferred into nucleofector cuvettes (Amaxa/Lonza). Nucleofection was performed in a Nucleofector II with the V-024 program. Cells were transferred to and maintained in complete RPMI 1640 medium at 37°C and used for analysis 72 h post-transfection. CD6 knockdown was confirmed by Western blotting, using MEM-98 42 for the detection of hCD6. Western blotting was performed as described previously 33. The membranes were then stripped and restained with an Ab specific to β-actin. The densities of bands were measured with a Quantity-One version 4.6.9 imaging system (BioRad) and normalized with β-actin.

### Cell-surface biotinylation and detection of biotinylated CD6

Cell-surface biotinylation and detection of biotinylated CD6 was performed as described previously 33.

### Tetramer production and staining

A cDNA fragment encoding the extracellular domain of human CD58 (residues Phe^29^-Arg^213^) was amplified by PCR from template kindly provided by M. Vuong (University of Oxford) using forward 5′-TAGGGATCCACCGGTTTTTCCCAACAAATATATGGT-3′ and reverse oligos 5′-CTAAGATCTGGATCCTCTTGAATGACCGCTGCTTGG-3′ containing *Age*I and *Bam*HI restriction sites, respectively. The CD58 fragment was cloned via *Age*I and *Bam*HI sites into the lab-modified TOPO vector containing the secretion peptide sequence (SP) from pHLsec 44 followed by a HA-tag with *Age*I and *Bam*HI sites immediately afterward. Fragments containing SP, HA-tag, and CD58 insert were then cut out from the TOPO vector constructs using *Hind*III and *Bam*HI for transfer into a lab-modified version of the pEE14 vector 45 using *Hind*III and *Bcl*I sites, in order to obtain CD58 chimeric cDNA encoding, in the following order, SP, HA-tag, the extracellular region of human CD58 (residues 29–213), and BirA 46 and 6×His tag sequences. The cDNA coding for the extracellular domain of hCD6 (residues Asp^25^-Glu^398^) was amplified from CD6/pBJ-neo 12 by PCR using forward 5′-TAGTAGGGATCCGACCAGCTCAACACCAGCAGTG-3′ and reverse 5′-CTACTAGGATCCTTAGTGATGTCTAGACTCCCGAGATTCCTTGTTC-3′ oligos containing *Bam*HI restriction sites. PCR products were purified and cloned into the *Bam*HI-*Bcl*I sites of the lab-modified version of pEE14, in order to obtain CD6 chimeric cDNA encoding, in the following order, SP, HA-tag, the extracelluar region of hCD6 (residues 25–398), and BirA and 6×His tag sequences.

The CD58 and CD6 chimera constructs were transfected into CHO-K1 cells using GeneJuice transfection reagent (Merck). Clones resistant to 25 μM methionine sulfoximine were selected 47 and screened for soluble CD58 (sCD58-BirA-His) or CD6 (sCD6-BirA-His) chimera expression using dot blots and Western blots. The best clones expressing sCD58-BirA-His or sCD6-BirA-His were selected for large-scale production of protein and grown in cell factories (Nunc). sCD58-BirA-His and sCD6-BirA-His secreted into tissue culture supernatants were harvested after approximately 4 wk and the proteins were extracted by metal-chelate chromatography using Ni-NTA agarose (QIAGEN). The sCD58-BirA-His and sCD6-BirA-His were eluted from the Ni-NTA agarose with 250 mM imidazole in 20 mM Tris-HCl, 0.5 M NaCl, pH 8.0, and further purified by size-exclusion chromatography. Soluble chimeras were then biotinylated using the BirA enzyme (Avidity LLC), and incubated overnight at room temperature with phycoerythrin (PE)-streptavidin (Invitrogen) at a 12:1 and 6:1 molar ratio, respectively, to allow the formation of tetrameric CD58 and CD6 complexes. The resulting tetramers were then purified using size-exclusion chromatography (Superose 6 HR 10/30 column) on an AKTA FPLC.

### Analysis of cytokine production and cell division

Jurkat E6.1 cells expressing hCD6 isoforms were plated at 10^5^ cells/well in complete RPMI, in the presence or absence of stimulation with OKT3 at 1 μg/mL. Supernatants were collected after 24 h and assayed for IL-2 production by ELISA (Abcam) according to the manufacturerŉs protocol.

Ag-induced cell proliferation was assessed via CFSE fluorescence. Human PBLs at 0.5×10^6^/mL were incubated with 1 μM CFSE (Molecular Probes) at 37°C for 15 min in PBS. For blocking the CD6–CD166 interaction, PBLs were then washed twice with PBS 20% FCS, and incubated with OX126 for 30 min at 4°C. During the same period of time, Raji cells (1×10^5^) were incubated with 3A6. The negative control Ab used was mouse IgG (BD Pharmingen). PBLs were stimulated by incubation with Raji cells prepulsed with a mix of sAgs. Cell division was determined by the extent of CFSE dilution detected by flow cytometry after 6 days of culture.

### Statistical analysis

Statistical analysis was performed using Graphpad Prism software (Version 5.04 for Windows, Graphpad Software, La Jolla, CA, USA). Studentŉs unpaired *t*-test was used to analyze continuous data; a χ^2^-test for independence was used to analyze noncontinuous data.

## Abbreviations

hCD166: human CD166
hCD6: human CD6
IS: immune synapse
MO: morpholino oligomer
rat CD6: rCD6
sAg: superantigen

## Supporting information

Detailed facts of importance to specialist readers are published as ”Supporting Information”. Such documents are peer-reviewed, but not copy-edited or typeset. They are made available as submitted by the authors.
