# Supplementary material for: CD6 attenuates early and late signaling events, setting thresholds for T-cell activation
Source: Eur J Immunol. 2011 Sep 28;42(1):195–205. doi: 10.1002/eji.201040528 (PMC3298641; doi:10.1002/eji.201040528)

European Journal of Immunology

Supporting Information

for

DOI 10.1002/eji.201040528

CD6 attenuates early and late signaling events, setting thresholds for T-cell activation

Marta I. Oliveira, Carine M. Gonçalves, Mafalda Pinto, Stéphanie Fabre, Ana Mafalda Santos, Simon F. Lee, Mónica A. A. Castro, Raquel J. Nunes, Rita R. Barbosa, Jane R. Parnes, Chao Yu, Simon J. Davis, Alexandra Moreira, Georges Bismuth and Alexandre M. Carmo

© 2011 WILEY-VCH Verlag GmbH & Co. KGaA, Weinheim

Supplemental Figure 1. Dot plots showing maximal calcium signals obtained in individual cells following stimulation by sAg-loaded Raji cells. **(A)** Dot plot of maximal calcium signals obtained in Fig. 3B. Each dot represents one cell. Horizontal lines indicate mean values [rCD6neg (n=15); rCD6+/rCD166 neg (n=11); rCD6+/rCD166+ (n=16)]. The probabilities that the calcium responses of rCD6+/rCD166 neg (p=0.0545) and rCD6+/rCD166+ (p=0.9506) T cells were similar to that of rCD6neg T cells to sAg were assessed using Student's t test. (B) Dot plot of maximal calcium signals from cells presented in Fig. 3G [rCD6neg (n=12); rCD6 d3 (n=16)]. The probability that the calcium response of rCD6 d3 T cells to sAg was similar to that of rCD6neg T cells was assessed using Student's t test (p=0.0847).

Supplemental Figure 2. Dot plots showing maximal calcium signals obtained in individual cells following stimulation by sAg-loaded Raji cells. Dot plot of maximal calcium signals obtained in Fig. 4B. Each dot represents one cell. Horizontal lines indicate mean values [rCD6CY5 (n=10); rCD6^{+} (n=15)]. The probability that the calcium response of rCD6+ T cells to sAg was similar to that of rCD6CY5 T cells was assessed using Student's t test (p=0.4882).

Oliveira et al. Suppl. Fig. 1

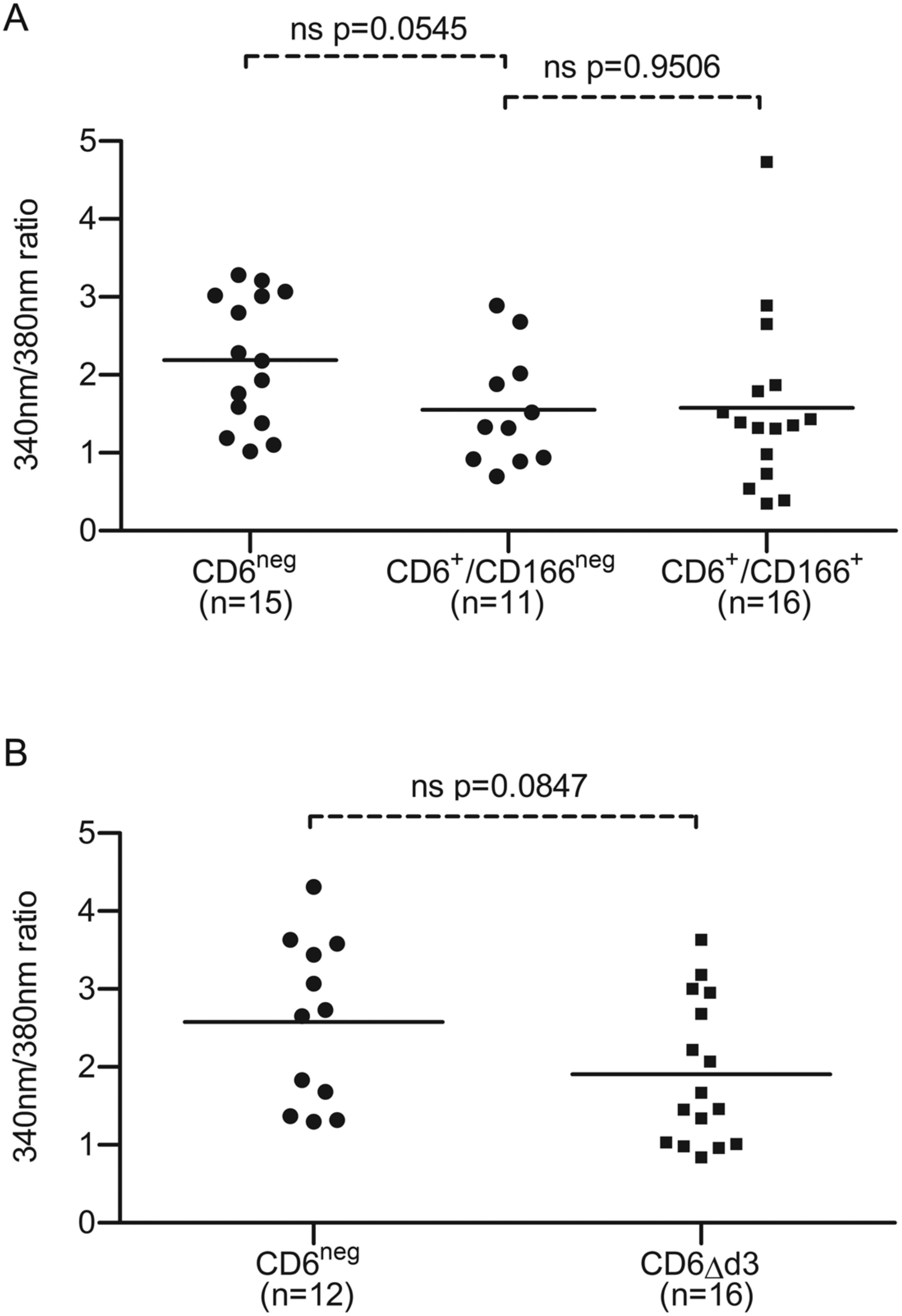

Oliveira et al. Suppl. Fig. 2

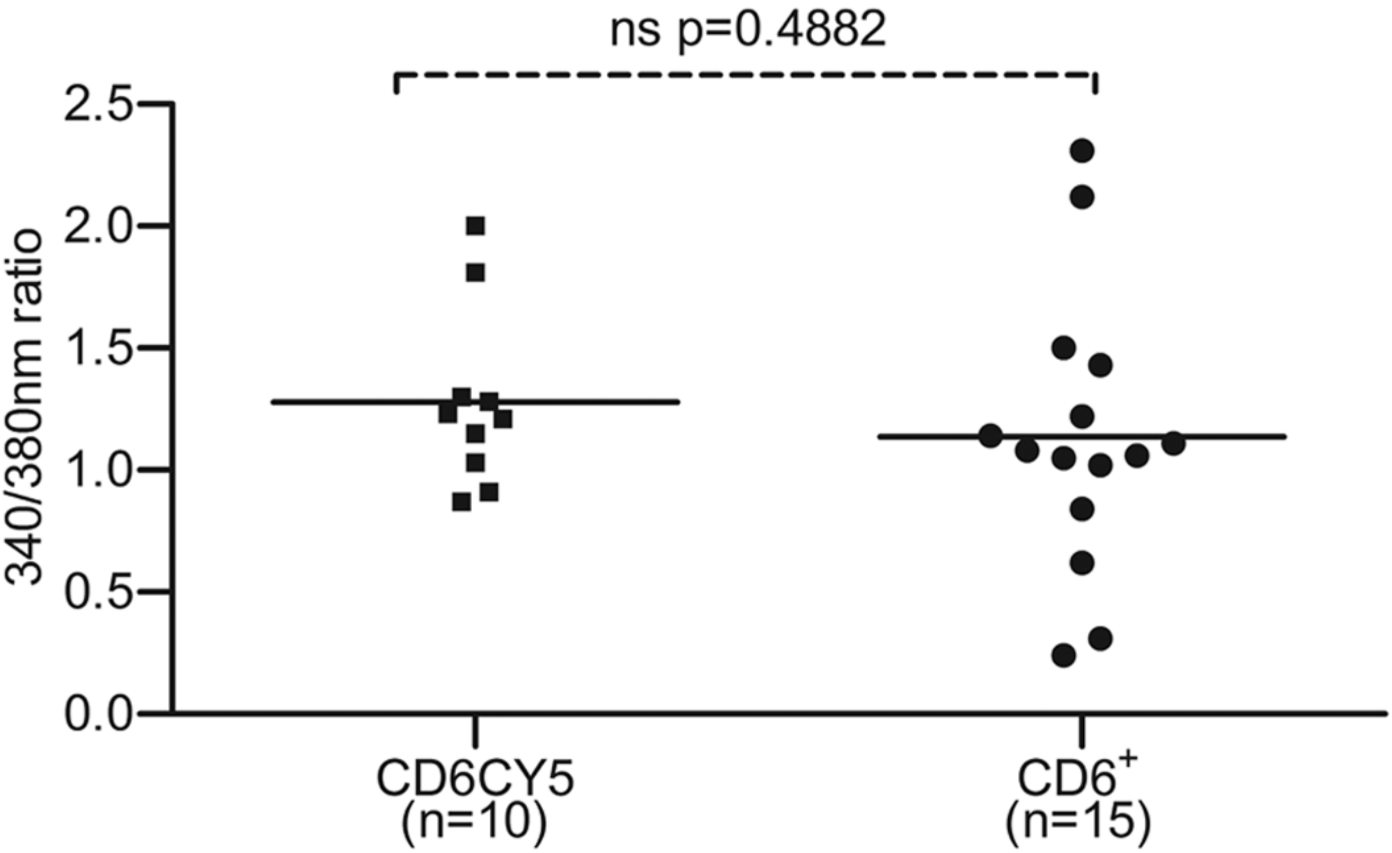

Supplement: Supplementary file 1 [file eji0042-0195-SD1.pdf]
